# Production of *LacZ* Inducible T Cell Hybridoma Specific for Human and Mouse gp100_25–33_ Peptides

**DOI:** 10.1371/journal.pone.0055583

**Published:** 2013-02-01

**Authors:** Gal Cafri, Adi Sharbi-Yunger, Esther Tzehoval, Lea Eisenbach

**Affiliations:** Department of Immunology, The Weizmann Institute of Science, Rehovot, Israel; University of Birmingham, United Kingdom

## Abstract

Identification and quantification of immunogenic peptides and tumor-derived epitopes presented on MHC-I molecules are essential for basic studies and vaccines generation. Although lymphocytes derived from transgenic mice can serve as sensitive detectors of processes of antigen presentation and recognition, they are not always available. The use of cell lines might be extremely useful. In this study, we generated a *lacZ* inducible CD8^+^ hybridoma (BUSA14) capable of recognizing both human and mouse gp100_25–33_ melanoma antigens presented on dendritic and tumor cell lines. This hybridoma expresses a variety of membranal T cell markers and secretes IL-2 and TNFα. Thus, BUSA14 offers a quantifiable, cheap and straightforward tool for studying peptide presentation by MHC-I molecules on the cell surface.

## Introduction

The key event in T cell activation is the recognition of a peptide bound to major histocompatibility complex (MHC) molecules on the surface of antigen presenting cells (APCs). The enormous pool of peptides displayed on MHC makes it almost impossible to detect a given peptide-MHC complex on the surface of APCs by using conventional indirect methods. On the other hand, direct recognition of a selected peptide by the TCR results in generation of intracellular signals leading to initiation of the primary stages of T cell activation [1]. To facilitate measurement of T cell activation and to enable identification of individual clones, β-galactosidase (*lacZ*) inducible CD4^+^ and CD8^+^ T cell hybrids were developed. Previous studies showed that heterologous *Escherichia coli* β-galactosidase (*lacZ*) gene, under control of the IL-2 entire enhancer region or the nuclear factor in activated T cells (NFAT) element alone, is specifically induced in transfected and activated T cells [2] [3] [4]. Thus, activation of transfected T cells, results in synthesis of both IL-2 and *lacZ* gene products. Moreover, since the *lacZ* remains sequestered within the activated cells, chromogenic or fluorogenic substrate enables measurement of an activating event in a single T cell [5] [3] [4]. Generation of the hybrids is relatively easy and allows maintenance in culture and the *lacZ* assay provides a rapid, sensitive and non-radioactive method for measuring T cell activation [1]. In this study we isolated T cells from Pmel-1 mice and generated a *lacZ* inducible CD8^+^ T cell hybridoma. The hybridoma possesses a TCR specific for the H-2D^b^ derived human and mouse gp100_25–33_ peptides, recognizes specific Ag-MHC complex on the surface of a dendritic cell line (DCs) or tumor cells, secretes T cell related cytokines and expresses a variety of membranal T cell markers.

## Materials and Methods

### Mice

Pmel-1 mice carry a rearranged T cell receptor transgene specific for the H-2D^b^ restricted, human gp100_25–33_ peptide [6] were originally purchased from the Jackson laboratory (Bar Harbor, ME, USA). Animals were maintained and treated according to the Weizmann Institute of Science and National Institute of Health guidelines. All experiments in mice were approved by the Institutional Use and Care Committee (IACUC) of the Weizmann Institute of Science.

### Cells

The OVA_257–264_–specific, H-2K^b^-restricted CTL hybridoma, B3Z [7], and the BWZ.36/CD8α fusion partner [2] were kindly provided by Dr. N. Shastri, University of California, Berkeley, USA. Cells were grown in lymphocyte medium containing RPMI 1640+HEPES (Invitrogen, Carlsbad, CA, USA), 10% FCS (HyClone, Bonn, Germany), 2 mM glutamine (Invitrogen), 1% Sodium pyruvate (Invitrogen), 1% non-essential amino acids (Invitrogen), 5×10^−5^ M β-mercaptoethanol (βME) and Penicillin-Streptomycin combined antibiotics. To avoid loss of CD8 expression, lymphocyte medium was supplemented with 1 mg/ml G418 (Invitrogen) for selection. Both B3Z and BWZ.36/CD8α harbor the NFAT-*lacZ* inducible reporter gene for T cell activation. The C57BL/6 (H-2^b^)-derived immortalized DC line DC2.4 [8] was kindly provided by Dr. K. Rock, UMass Medical School, North Worcester, MA, USA. DC2.4 cells were cultured in RPMI 1640 medium supplemented with 10% FCS, 2 mM L-glutamine and combined antibiotics. The C57Bl/6 derived, highly metastatic, poorly immunogenic, low MHC class-I expressing cell line B16-F10.9 (F10.9) [9], the high-metastatic, low-immunogenic D122 clone of the 3LL carcinoma, of C57BL/6 (H-2^b^) origin and the carcinogen-induced T cell lymphoma EL4 cells (H-2^b^) were grown in DMEM (Invitrogen) containing 10% FCS, 2 mM L-glutamine, 1 mM sodium pyruvate, 1% nonessential amino acids and 1% Penicillin-Streptomycin combined antibiotics. The C57Bl/6 derived, chicken Ovalbumin transfected, highly metastatic B16-MO5 [10] cell line was cultured in B16-F10.9 medium supplemented with 2 mg/ml G-418. Both F10.9 and B16-MO5 over express the tumor associated murine gp100 protein.

### Generation of T cell hybridomas

Total splenocytes were isolated from spleens of Pmel-1 mice. Cells were washed once with PBS and resuspended in 6 ml OptiMEM medium (Invitrogen). Four ml were transferred into flasks containing 40 ml of lymphocyte medium and incubated at 37°C. As sensitizing cells, Two ml were incubated with 30 µg/ml hgp100_25–33_ peptide for 2 hours, diluted in 10 ml lymphocyte medium and added to the flasks. Four days later, Cells were washed once with PBS, separated on Lympholyte-M (Cedarlane, Burlington, NC, USA) and fused with the BWZ.36/CD8α cells using polyethylene glycol (PEG1500; Boehringer Mannhiem, Indianapolis, IN, USA) as described before [1]. Briefly, equal numbers (10×10^6^) of lymphocytes and BWZ.36/CD8α cells were mixed in a 50 ml conical centrifuge tube and washed once in pre-warmed serum-free RPMI 1640 medium. The supernatant was aspirated and the pellet was loosened by gentle tapping. One ml of 50% PEG was slowly added during 90 seconds. The PEG was then diluted with 10 ml warm serum-free medium and the tube was placed in a 37°C water bath for 8 minutes. Then, cells were centrifuged, resuspended in lymphocyte medium to 3×10^5^/ml, and added (0.1 ml) to each well of 96 well plates. Twenty-four hours later, HAT and hygromycin were added (final concentrations of 1.36 mg/ml hypoxanthine (Sigma), 17.6 mg/ml aminopterin (Sigma), 388 mg/ml thymidine (Sigma), and 400 U/ml hygromycin (Invitrogen). Resistant clones were observed starting 10–15 days later. All clones were tested for antigen recognition in T cell activation assay as described in section 2.6.

### Antibodies and peptides

The anti mouse APC-CD279, APC-CD44, FITC-CD69, FITC-CD8, FITC-CD62L, PerCp-Cy5.5-IFNγ, eFluor450-TNFα, PE-IL-4, PE-TCRVβ13, PE-Cy7-CD8 and AlexaFluor 488-CD107a were all purchased from eBioscience (San Diego, CA, USA). Antibodies against mouse CTLA4 and FcγII/III receptors (2.4G2 hybridoma, Fc Block) were purchased from BioXcell (West Lebanon, NH, USA). The APC conjugated anti mouse IL-2 was purchased from BD (Becton Dickinson, San Jose, CA, USA). The FITC-conjugated F(ab′)_2_ fragment donkey anti-mouse IgG (H+L) was purchased from Jackson ImmunoResearch Laboratories (West Grove, PA, USA). The OVA_257–264_ (SIINFEKL), human gp100_25–33_ (KVPRNQDWL) and mouse gp100_25–33_ (EGSRNQDWL) peptides were synthesized by Sigma-Aldrich (Rehovot, Israel).

### Flow cytometry

#### Indirect staining

One million cells were harvested, washed twice using 3 ml FACS buffer (0.5% BSA, 0.1% sodium azide in PBS) and incubated with 1 µg/ml of primary antibody (Ab) for 1 hour at 4°C. Samples were washed twice with FACS buffer and stained with 1 µg/ml of FITC-labeled secondary Ab for 1 hour at 4°C. Then washed twice, resuspended in 0.5 ml cold PBS with 0.1% sodium azide and analyzed.

#### Direct staining

Cells were harvested, washed once with cold FACS buffer, and incubated for 30 minutes at 4°C in the dark with antibodies (at the concentrations recommended by the manufacturer). Cells were incubated for and washed once using 3 ml FACS buffer, resuspended in 0.5 ml PBS with 0.1% sodium azide and analyzed by flow cytometry.

#### Intracellular cytokine staining

DC2.4 cells, 2.5×10^6^, were harvested and incubated with the relevant peptides at 10 µg/ml in OptiMEM for 1 hour. Then, 6×10^5^ peptide loaded DC2.4 and 2×10^6^ hybridoma cells were added to a 24 well plate followed by centrifugation at 1000 rpm for 5 minutes at 18°C. As positive control for hybridoma activation, 50 ng/ml PMA (Phorbol 12-Myristate 13-Acetate, Sigma) and 750 ng/ml ionomycin (Sigma) were add to some wells. The plates were incubated for 2 hrs at 37°C, 5% CO2. Brefeldin A (BFA, eBioscience), at a final concentration of 3 µg/ml was added to all wells and the plates were centrifuged at 500 rpm, 5 min at 18°C. Following incubation for 4 additional hours, cultures were harvested, washed with staining buffer and fixated with 0.1 ml fixation buffer (PBS w/o Ca and Mg+4% paraformaldehyde) at 4°C for 20 minutes. Then washed twice and resuspended in 1 ml permeabilizing solution (PBS (w/o Ca and Mg supplemented with 0.1% Saponin, 5% FCS and 0.1% azide) for 15 minutes at 4°C. Samples were then washed and stained for cell surface markers and intracellular cytokines. All samples were analyzed by SORP LSRII (Becton Dickinson, San Jose, CA, USA) and FlowJo software (ThreeStar, San Carlos, CA, USA).

### T cell hybridoma activation assay

**A.** DC2.4 cells were loaded with varying concentrations (0.001–100 µg/ml) of human or mouse gp100_25–33_. Culture were set in 96-well plates by adding equal numbers (6×10^4^) of peptide-loaded DC2.4 and hybridoma cells for 12 hours.

**B.** DC2.4 cells were loaded with 50 µg/ml hgp100_25–33_ or control peptide. Cultures were set in 96-well plates by mixing 6×10^4^ of peptide-loaded DC2.4 and 4.7×10^2^–6×10^4^ hybridoma cells for 12 hours.

For both assays, growth medium was removed and cells were washed once with 100 µl PBS. For lysis, 100 µl of lysis buffer (PBS with 9 mM MgCl_2_, 0.125% NP40) containing 0.3 mM chlorophenol red β-D galactopyranoside (CPRG (Sigma) were added to each well, mixed, and clear lysates were transferred into new 96 well plates. One to twenty four hours later, the optical density of each well was detected with a Synergy HT Multi-Mode Microplate reader (BioTek Winooski, VT, USA) at 570 nm using 630 nm as reference.

### Cytotoxicity assays

Hybridomas were washed and incubated for 4 hours with L-[^35^S]methionine (PerkinElmer, Waltham, MA, USA) labelled target cells at different Effector∶Target ratios [11]. The percentage of specific lysis of triplicates was calculated as follows: (average experimental cpm - average spontaneous cpm)/(average maximum cpm - average spontaneous cpm)×100. Maximal L-[^35^S]methionine containing protein release was obtained by lysis of target cells with 0.1 M NaOH.

## Results

### Generation of human and mouse gp100_25–33_ specific T cell hybridoma

**Figure 1:**
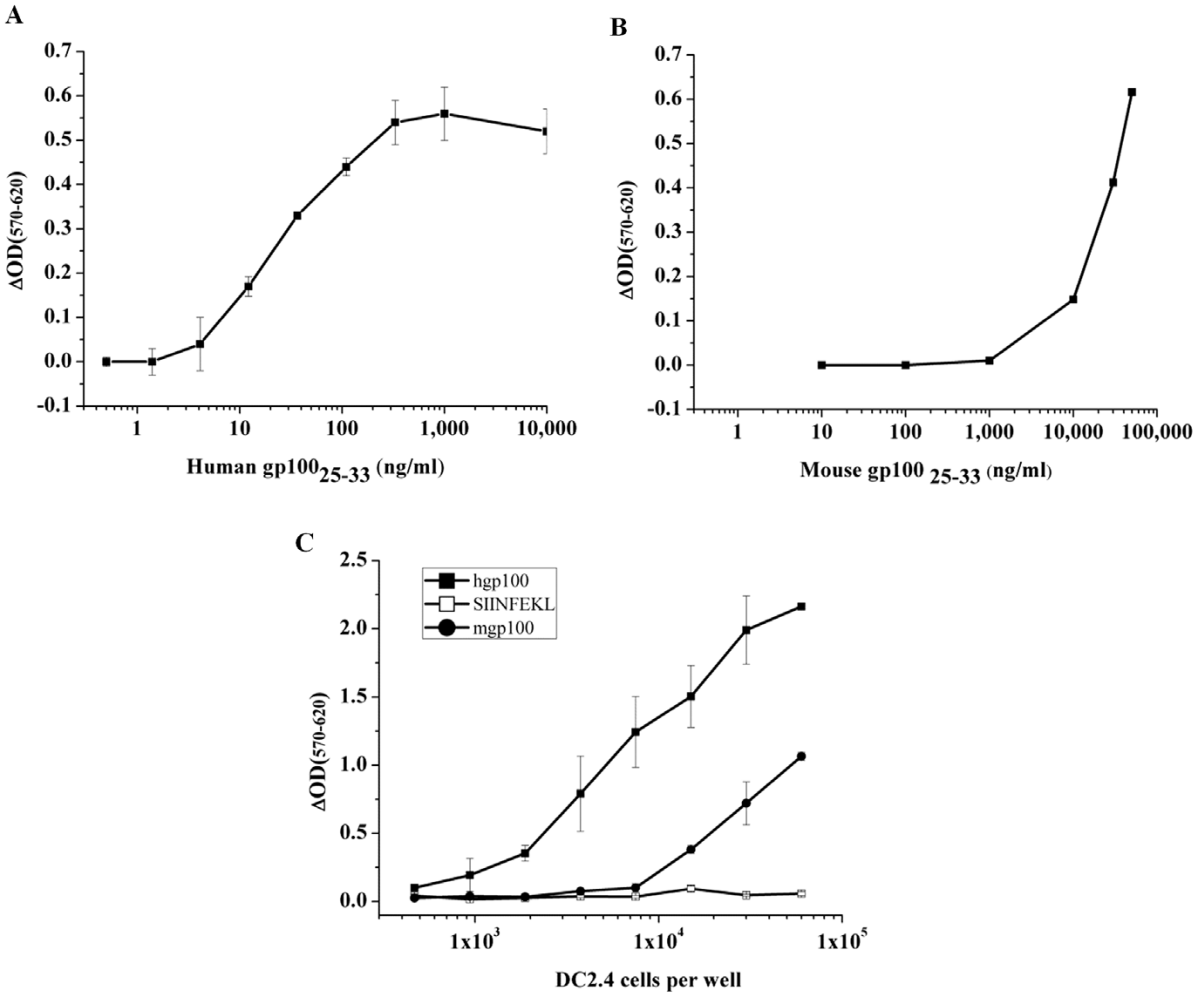
BUSA14 cells are efficiently activated by mouse and human gp100_25–33_ peptides. DC2.4 cells were loaded with hgp100_25–33_ at doses ranging from 0.5 to 10000 ng/ml (**A**) or mgp100_25–33_ (10 to 50000 ng/ml)(**B**). Then co-cultured with BUSA14 for 12 hours. Cells were then lysed and β-Gal enzymatic activity was detected with CPRG. Representative results (1 of 3 experiments) are presented as ΔOD (sample OD-background OD) measured after 3 hours. **C.** Sixty thousand BUSA14 cells/well were incubated in triplicate overnight with serial dilutions of DC2.4 cells loaded with 50 µg/ml hgp100_25–33_ or mgp100_25–33_. SIINFEKL loaded DC2.4 cells were used as negative control. Assays were done as before and presented as ΔOD measured after 24 hours.

In order to produce *LacZ* inducible T cell hybrids, specific to human and mouse gp100_25–33_ peptides, we took advantage of CD8+ T cells isolated from Pmel-1 mice [6], which carry a transgenic TCR specific for the Pmel17/gp100 derived, H-2D^b^ restricted gp100_25–33_ peptide. Lymphocytes isolated from Pmel-1 mice were activated in-vitro by peptide and fused with the BWZ.36/CD8α cells [2] harboring the NFAT-*lacZ* inducible reporter gene for T cell activation. After drug selection, hybrids were subjected to screening assays in order to identify hgp100_25–33_ specific clones (data not shown). Few positive clones were detected and one of them, named BUSA14, was selected for further functional and morphological characterizations. First, we measured the antigen induced *LacZ* response to mouse and human gp100_25–33_ peptides. As APC we used DC2.4 [8], a C57BL/6 derived immortalized DC line, which expresses both H-2K^b^ and H-2D^b^ molecules at high levels on the cell surface, thus allowing efficient peptide loading and presentation. DC2.4 cells were loaded with varying amounts of mouse or human gp100_25–33_ and subjected to hybridoma activation assays with BUSA14 cells. As shown in Fig. 1A and B, both mouse and human gp100_25–33_ loaded DC2.4 cells elicited activation of BUSA14 cells. Major differences can be seen in the dose response to the two peptides, presumably as a result of the increased affinity of hgp100_25–33_ to H-2D^b^ molecules, allowing long lasting complexes on the cell surface of DC2.4 cells. In another experiment, serial dilutions of DC2.4 cells, pre- loaded with 50 µg/ml mouse or human gp100_25–33_, were incubated with a constant number of BUSA14 cells. As shown in Fig. 1C, hgp100_25–33_ loaded DC2.4 cells are by far more efficient in activating BUSA14 cells.

### Surface molecules expressed by resting and activated BUSA14 cells

**Figure 2:**
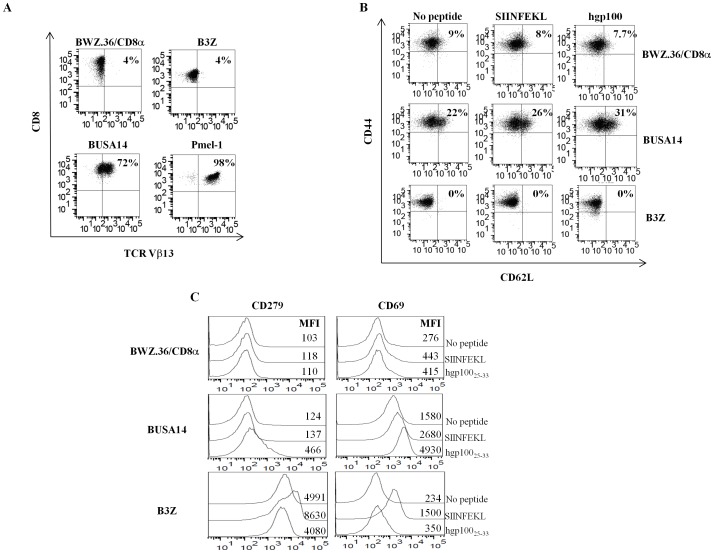
T cell markers expressed by BUSA14 cells. **A.** BUSA14, B3Z and BWZ.36/CD8α cells were analyzed by flow cytometry and monoclonal antibodies against CD8 and TCR Vβ13 to confirm the Pmel-1 TCR expression. **B.** BUSA14, BWZ.36/CD8α and B3Z cells were co-cultured with hgp100_25–33_ or SIINFEKL for 12 hours, or remained untreated. Cells were stained with antibodies to CD8, CD62L and CD44 and analyzed by flow cytometry. Cells were gated for CD8 to exclude DC2.4 cells. **C.** Two million BUSA14, BWZ.36/CD8α and B3Z cells were co-cultured with 6×10^5^ DC2.4 loaded with hgp100_25–33_ or SIINFEKL for 12 hours, or remained untreated. Then stained with antibodies against CD8, CD69, CD279 and analyzed by flow cytometry. Cells were gated for CD8 to exclude DC2.4 cells. Mean fluorescent intensity (MFI) values are presented in the figure. This figure is a representative of three experimental repeats.

To further characterize the generated hybridoma, cells were analyzed for surface expression of CD8 and the Pmel-1 specific TCR Vβ13 chain. The results presented in Fig. 2A clearly indicate that only BUSA14 and not B3Z or BWZ.36/CD8α express TCRVβ13. In order to test whether restimulation with hgp100_25–33_ promotes the differentiation of BUSA14 cells towards later activation stages, we performed an activation assay. DC2.4 cells were loaded with hgp100_25–33_ and co-incubated for 12 hours with BUSA14, B3Z or BWZ.36/CD8α cells. The mixed cultures were analyzed by flow cytometry for surface expression of CD69, CD279, CD62L and CD44. As shown in Fig. 2B, all 3 hybridomas retained their effector phenotype (CD44^hi^/CD62L^low^) following peptide stimulation. As shown in Fig. 2C, CD69 levels were up-regulated both in activated BUSA14 and B3Z cells while CD279 up-regulation occurred only in B3Z cells.

### Detection of cytokines produced by resting and activated BUSA14 cells

**Figure 3:**
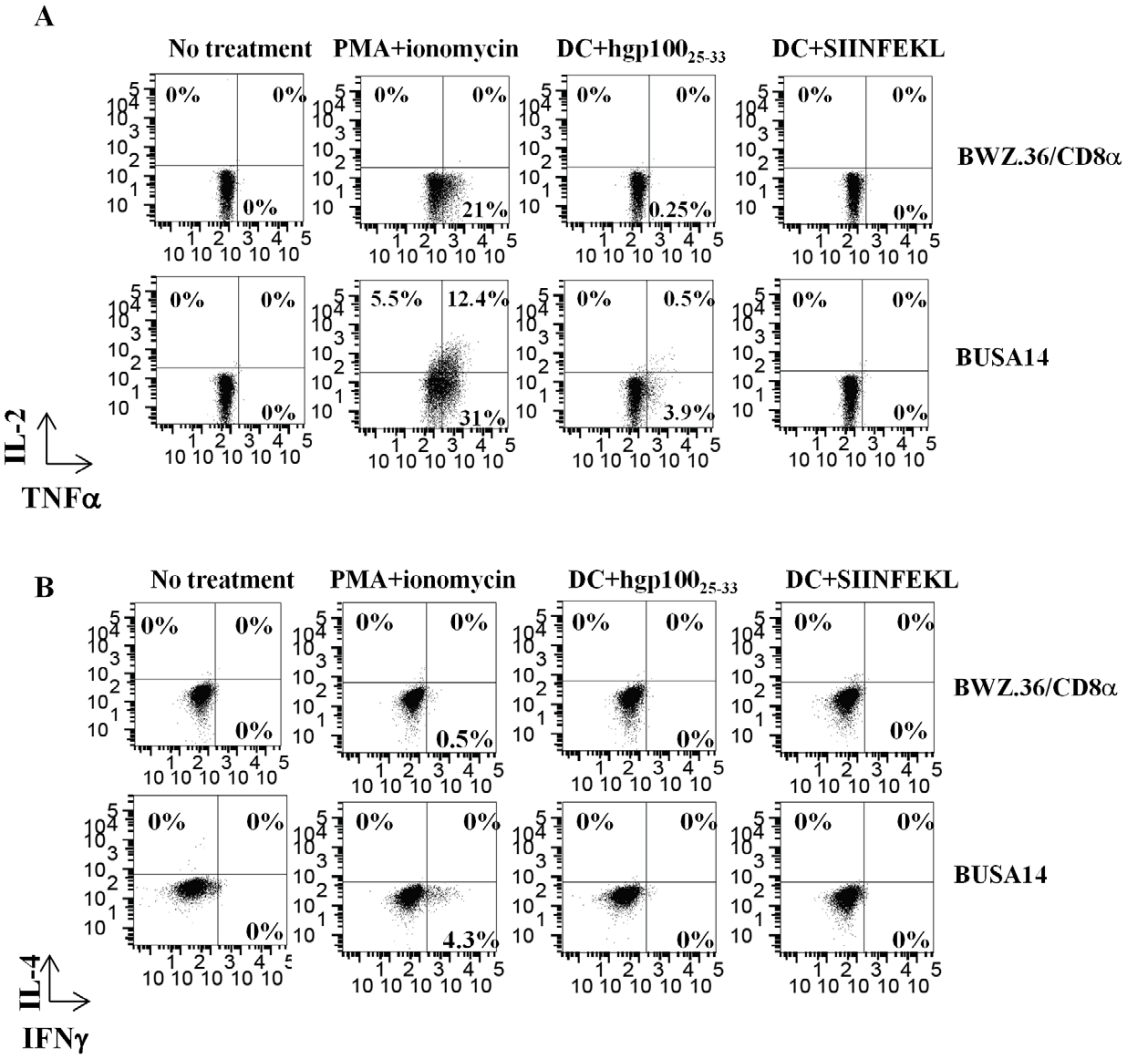
Detection of cytokines produced by BUSA14 cells. BUSA14 or BWZ.36/CD8α were co-incubated with DC2.4 cells loaded with hgp100_25–33_ or SIINFEKL. Cells alone or co-cultured with unloaded DC2.4 or with PMA and ionomycin served as negative and positive controls, respectively. All cells were intracellulary stained with antibodies to CD8, IL-2/TNFα (**A**), IL-4/IFNγ (**B**) and analyzed by flow cytometry. Cells were gated for CD8 to exclude DC2.4 cells.

BUSA14 and BWZ.36/CD8α Cells were co-incubated with DC2.4 loaded with hgp100_25–33_ or SIINFEKL for 6 hours and intracellular stained with antibodies against CD8, IL-2, IL-4, TNFα, IFNγ and CD107a. Cells co-cultured with unloaded DC2.4 or PMA and ionomycin served as negative and positive controls, respectively. As shown in Fig. 3, BUSA14 cells produced a variety of cytokines following activation with PMA and ionomycin. Thirty percent of BUSA14 cells produced TNFα, 12% produced TNFα and IL-2, 6% and 4% produced IL-2 and IFNγ respectively. Following activation with hgp100_25–33_ loaded DC2.4, only 4% of the cells generated low amounts of TNFα. BWZ.36/CD8α cells showed moderate TNFα expression only following activation by PMA and ionomycin. We could not detect CD107a on BUSA14 or BWZ.36/CD8α following incubation in presence of peptide or PMA (data not shown).

### Activation of BUSA14 cells did not result in cytotoxic capacity

**Figure 4:**
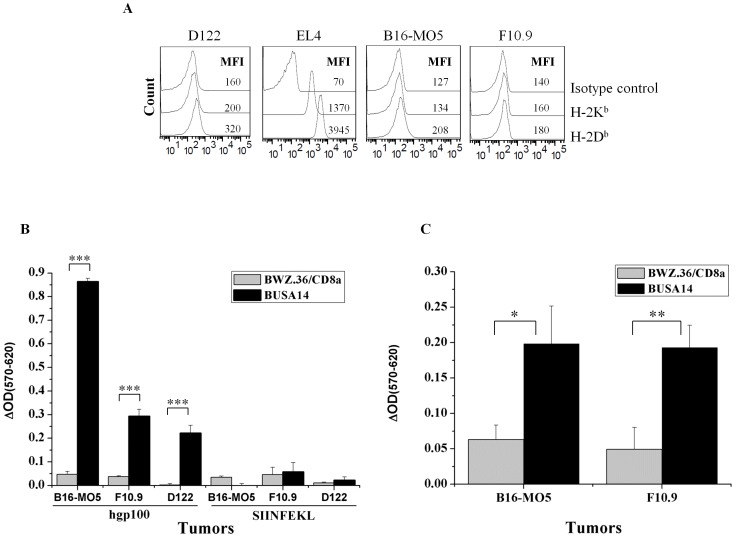
BUSA14 are activated by hgp100_25–33_ and mgp100_25–33_ presented on melanoma cell lines. **A.** B16-MO5, F10.9, D122 and EL4 tumor cell lines were analyzed by flow cytometry with monoclonal antibodies to H-2K^b^ and H-2D^b^ to analyze MHC-I membranal expression. MFI values are presented in the figure. **B.** Twenty thousand B16-MO5, F10.9 and D122 cells were loaded with 30 µg/ml hgp100_25–33_ or SIINFEKL peptides. Cells were washed and co-incubated with 6×10^4^ BUSA14 and BWZ.36/CD8α for 12 hours. Cells were then lysed and β-Gal enzymatic activity was monitored with CPRG. Cultures with D122 served as reference for CPRG background levels. Representative results (1 of 3 experiments) are presented as ΔOD (sample OD-background OD) measured after 12 hours. **C.** Sixty thousand BUSA14 and BWZ.36/CD8α cells/well were incubated overnight, in triplicates, with 2×10^4^ B16-MO5, F10.9 or D122 tumor cell lines. Representative results (1 of 2 experiments) are presented as ΔOD (sample OD-background OD) measured after 24 hours. Statistical analysis was done using student T test (*p<0.05, **p<0.01, ***p<0.001).

Aiming at further investigating whether BUSA14 cells are activated following presentation of mgp100 by melanoma cells, we incubated the hybrids with F10.9 or B16-MO5. Co-culturing with D122 clone of 3LL lung carcinoma served as negative control. Since gp100_25–33_ is presented on H-2D^b^, these tumor lines were analyzed for membranal MHC-I (Fig. 4A, EL4 cells served as positive control). In another experiment, the tumor cells were loaded with hgp100_25–33_ or SIINFEKL before co-culturing with BUSA14 or BWZ.36/CD8α As shown in Fig. 4B, incubation in presence of all three hgp100_25–33_ loaded tumor lines resulted in activation of BUSA14 as detected by CPRG assays. We than tested whether BUSA14 cells are activated by the endogenously processed mgp100_25–33_ peptide on the surface of B16-MO5 and F10.9 melanoma lines. CPRG assays were done following 12 hours of culturing. Co-incubation with D122 cells served as reference for CPRG background levels. As shown in Fig. 4C, BUSA14 cells recognized the mgp100_25–33_ on the surface of both B16-MO5 and F10.9 tumor lines. Although BUSA14 cells were activated by mgp100_25–33_ presented on melanoma lines, we could not detect any killing of these cells by BUSA hybrids (data not shown).

## Discussion

In this study we generated a *LacZ* inducible T cell hybridoma specific for mouse and human gp100_25–33_ peptides. The hybridoma, named BUSA14, specifically recognize peptide-MHC class I complexes and is specifically activated by APCs and tumor cell lines presenting the gp100_25–33_ peptide. BUSA14 expresses cell surface markers similar to the set expressed on activated T cells. Although activated by melanoma lines, this hybridoma did not exhibit cytotoxic activity against these tumors.

The different affinities of mgp100_25–33_ and hgp100_25–33_ to H-2D^b^ molecules can be used to study MHC-TCR interactions of low and high affinity peptides using the BUSA14 cells. The fact that BUSA14 can recognize the mgp100_25–33_ on the surface of tumor cells can offer an easy and accurate system for screening tumor lines for peptide presentation, or to study escape mechanisms involving MHC down regulation and inhibition of antigen processing in tumor cells.

To summarize, we offer a peptide specific hybridoma that is straightforward, sensitive, accurate and easy to maintain. It is advantageous when compared to other cellular assays designed for measuring peptide presentation that are expensive, time consuming and require primary cells. BUSA14 cells can serve as a highly applicable tool for studying TCR-MHC interactions at both high and low affinity peptide systems.
